# The efficacy of gabapentin for the treatment of refractory cough associated with interstitial lung disease: study protocol for a randomized, double-blind and placebo-controlled clinical trial

**DOI:** 10.1186/s13063-022-06059-5

**Published:** 2022-02-21

**Authors:** Ronglin Gao, Xianghuai Xu, Shengyuan Wang, Jincheng Pu, Cuiqin Shi, Siwan Wen, Yiqing Zhu, Jianping Tang, Xuan Wang, Li Yu

**Affiliations:** 1 Department of Rheumatology and Immunology, Tongji Hospital, School of Medicine, Tongji University, No. 389 Xincun Road, Shanghai, 200065 China; 2 Department of Pulmonary and Critical Care Medicine, Tongji Hospital, School of Medicine, Tongji University, No. 389 Xincun Road, Shanghai, 200065 China

**Keywords:** Gabapentin, Interstitial lung disease, Refractory cough, Cough hypersensitivity syndrome

## Abstract

**Introduction:**

Gabapentin, a neurotransmitter modulator, is thought to treat refractory cough associated with interstitial lung disease by improving cough hypersensitivity.

**Methods/design:**

This is a single-center, prospective, randomized, double-blind, placebo-controlled trial. The trial will investigate the effect of a 10-week course of oral gabapentin 900 mg/day on refractory cough associated with interstitial lung disease (ILD) and explore the possible mechanisms involved in improving cough symptoms. A total of 84 individuals will be randomized in a 1:1 ratio to two treatment groups and will be followed for a total of 14 weeks from the first dose. The primary endpoint of the study will be the change in cough symptom scores at 14 weeks. The secondary endpoints include the change in Leicester Quality of Life Questionnaire (LCQ), Gastroesophageal Reflux Disease Questionnaire (Gerd Q), and Hull Airway Reflux Questionnaire (HARQ) scores; cough sensitivity (C2 and C5) values; and safety.

**Discussion:**

This study will be the first randomized, controlled clinical trial to investigate gabapentin for the treatment of refractory cough associated with interstitial lung disease and provide data on efficacy, safety, and quality of life. If the study confirms that gabapentin is effective in improving refractory cough associated with interstitial lung disease, it will indicate that a deeper understanding of its mechanisms may reveal new therapeutic targets.

**Trial registration:**

Chinese Clinical Trial Registry ChiCTR2100045202. Registered on 8 April 2021, www.chictr.org.cn

**Supplementary Information:**

The online version contains supplementary material available at 10.1186/s13063-022-06059-5.

## Background

Interstitial lung disease (ILD) is a collective term for a group of acute and chronic lung diseases that affect the airways, lung parenchyma, and pulmonary vascular system, with varying degrees of airway inflammation and fibrosis. It is categorized either as disease with a clear etiology or of unknown cause. The former mainly includes ILD caused by connective tissue disease (CTD), environmental exposure, drugs, and other factors, while ILD of unknown cause includes seven main types, the most common of which is idiopathic pulmonary fibrosis (IPF). Although the etiology differs, the symptoms of ILD are common to both categories and include dyspnea, cough, pulmonary hypertension, and difficulty sleeping. Cough is the most common symptom and is the most resistant to treatment, and it may even be the main manifestation in some ILDs. One study found that up to 80% of patients with ILD, including CTD-ILD and IPF, had chronic cough [1]. Focusing on the assessment of cough associated with IPF, scholars have established various tools, such as 24-h cough monitoring and health-related quality of life questionnaires [2]. ILD-related cough is significantly detrimental to health-related quality of life, in common with chronic cough of unknown etiology [2, 3], and plays an important role in the mental health of patients with autoimmune disease. It is extremely important to seek new treatments for chronic cough in ILD patients, concurrently controlling the airway inflammation and lung fibrosis.

The available studies on ILD-related cough are relatively few and not rigorous. They indicate that some drugs may be effective, but their specific mechanisms are unknown, the incidence of adverse drug events is high, and their targets are unclear. Therefore, further studies remain necessary. Pirfenidone and nintedanib are used in the anti-fibrosis treatment of ILD [4]. An uncontrolled study showed that this form of treatment may reduce the severity of cough [5], but this finding has not been supported by larger clinical trials. An open-label study of 6 patients conducted by Hope-Gill and others found that corticosteroids had the effect of reducing cough [6]; however, the “triple therapy” of corticosteroids combined with azathioprine and n-acetylcysteine in the treatment of IPF would lead to an increase in mortality compared with a placebo group [4]. Therefore, the application of corticosteroids should be limited to patients at risk of severe ILD or patients who also have asthma or eosinophilic bronchitis. A single-center study of thalidomide in the treatment of IPF-related cough including only a few patients showed that thalidomide could significantly improve patients’ quality of life [7], via a mechanism of action thought to be related to anti-inflammatory effects and possible reduction of cough sensory nerve activity. However, thalidomide’s significant side effects are contraindications for IPF-related coughs.

The mechanisms underlying cough in patients with ILD are complex and diverse. They may be related to airway inflammation, sensitization, or pulmonary fibrosis and present significant challenges in the search for the cause of cough and its treatment in ILD patients. ILD-related cough may be due to mechanical distortions associated with pulmonary parenchymal fibers [8], and elevated cough sensitivity, airway inflammation, ILD medication, infection, and co-morbidities (e.g., gastroesophageal reflux, upper respiratory disease, and asthma) may also be potentially important mechanisms [9–11]. Multiple clinical studies have shown that cough symptoms persist in ILD patients while on anti-inflammatory and anti-fibrotic therapy, suggesting that improving airway sensitivity may be an avenue for treatment of cough symptoms in ILD patients.

Studies show that patients with chronic cough have common clinical features of increased cough sensitivity. Therefore, Morice et al. proposed the concept of cough hypersensitivity syndrome (CHS), in which chronic cough is the only or prominent symptom of cough hypersensitivity [12]. Based on this concept, re-establishing normal cough sensitivity may be an important strategy for the future treatment of refractory cough. While treating the etiology of cough is important, correcting the pathophysiology of abnormal cough hypersensitivity also deserves attention, especially for refractory cough, in which effective treatment is lacking and modulation of cough sensitivity is particularly important [13]. The concept of CHS may indicate new research directions for the development of more effective therapeutic agents and future improvement of the diagnosis and treatment of refractory cough, as acknowledged in relevant literature [14].

Gabapentin acts as a neuromodulator by specifically binding to the α2δ subunit of voltage-gated calcium channels in the brain and is primarily used for the treatment of epilepsy and neuropathic pain. Because patients with chronic cough have a central hypersensitivity similar to that of neuropathic pain [15], gabapentin is also used to treat refractory chronic cough [16]. Well-designed clinical trials have demonstrated that gabapentin at doses ranging from 300 to 1800 mg/day for 8 weeks significantly improves symptoms and quality of life in patients with refractory cough [17]. Since ILD-related refractory cough may be associated with airway cough sensory nerve hypersensitivity, we hypothesize that gabapentin may have good efficacy in this condition. There is limited evidence to support the treatment of chronic cough in ILD. For refractory or unexplained cough in ILD, the guideline recommends gabapentin, which has been shown to reduce the severity and frequency of cough and improve quality of life in patients with refractory cough in one trial, although further clinical studies are needed to validate this [18]. Gabapentin is also recommended for the treatment of refractory cough in both the new Chinese and US cough guidelines [19, 20]. Our previous study and other studies have shown that the effectiveness of gabapentin in the treatment of refractory cough is around 57% [21, 22]. Additionally, gastroesophageal reflux (GER) is one of the main causes of cough in patients with ILD and 36% of gastroesophageal reflux disease (GERD) is refractory. Madanick et al. combined 300 mg–900 mg/day of gabapentin with a control drug as a treatment for GERD and found at least 50% reduction in cough symptoms in 75% of patients [23]. Our previous clinical study also confirmed that gabapentin as a supplement to standard anti-reflux drug therapy was effective in controlling cough in 57% of refractory gastroesophageal reflux cough. The curative effect is similar to that of baclofen, another neuro factor modulator with fewer side effects, and is better tolerated by patients, and it usually takes effect within 1 week [22]. It has also been confirmed that fast-acting treatment may be achieved by the administration of anti-reflux measures containing gabapentin at the beginning of treatment. These findings suggest that gabapentin is a good clinical option for the treatment of refractory cough caused by ILD.

### Aims and objectives

1. To investigate whether gabapentin improves cough symptoms in patients with ILD

2. To explore the potential mechanism of cough symptom alleviation in gabapentin treatment for ILD

## Methods/design

**Fig. 1:**
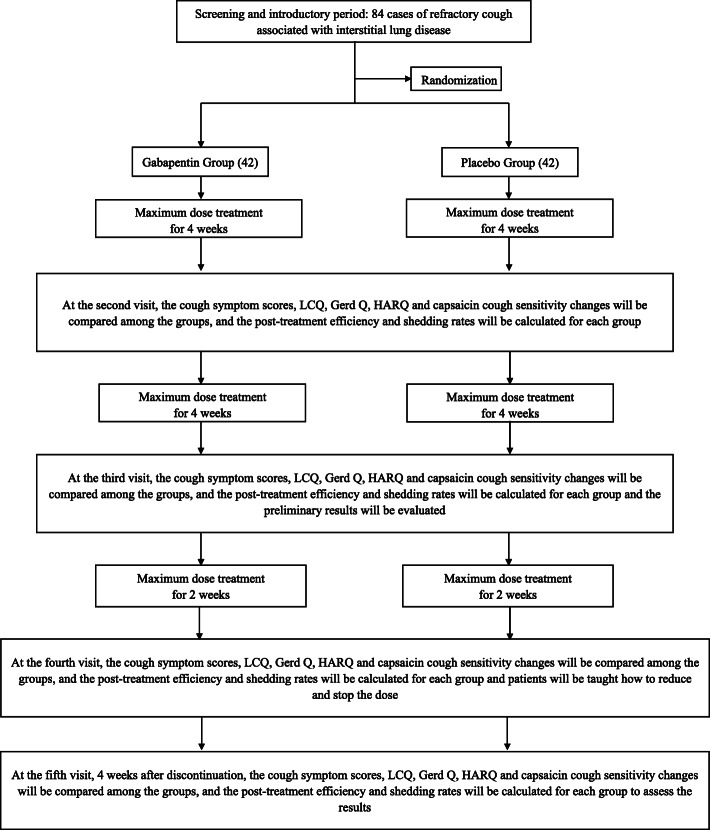
Flowchart of the study design. Abbreviations: LCQ, Leicester Quality of Life Questionnaire; Gerd Q, Gastroesophageal Reflux Disease Questionnaire; HARQ, Hull Airway Reflux Questionnaire

The study is a single-center, prospective, randomized, double-blind, placebo-controlled trial, designed to identify the impact of gabapentin in improving cough in ILD. Patients will be randomized into two groups. The schedule of events for the enrolment, interventions, and assessments of participants is shown in Fig. 1 and Table 1.

**Table 1:** Outline of the planned visit

	Study period						
	**Screening visit**	**Randomize**	**Treatment visits**				**Post-treatment follow-up**
Timepoint**	***−7~0***		**Visit 1** **Day 0**	**Visit 2** **Week 4**	**Visit 3** **Week 8**	**Visit 4** **Week 10**	**Visit 5** **Week 14**
**Eligibility screen**	X						
**Informed consent**	X						
**Adverse event check**			X	X	X	X	X
**Study drug**			X	X	X	X	
**Concomitant medication**			X	X	X	X	X
**Assessments**							
**Physical exam**	X						
**Cough symptom score**	X		X	X	X	X	X
**LCQ**	X		X	X	X	X	X
**Gerd Q**	X		X	X	X	X	X
**HARQ**	X		X	X	X	X	X
**Capsaicin cough sensitivity**	X		X	X	X	X	X
**Induced sputum analysis**	X		X	X	X	X	X
**Pulmonary function**	X		X				X
**Chest CT**	X		X				X

*LCQ*, Leicester Quality of Life Questionnaire, *Gerd Q*, Gastroesophageal Reflux Disease Questionnaire, *HARQ*, Hull Airway Reflux Questionnaire, *Chest CT*, chest computer tomography

### Location and setting

The study will recruit subjects from Shanghai Tongji Hospital, School of Medicine, Tongji University. The trial was reviewed by the ethics committee of Shanghai Tongji Hospital, no. (2021-006), and registered on the Chinese Clinical Trial Registry, ID ChiCTR2100045202.

### Participant timeline

Screening and introductory period (day −7~0): 0–7 days from baseline; each patient will be asked to sign an informed consent form (ICF); eligibility will be checked based on inclusion/exclusion criteria; patient demographic data and medical and medication history will be gathered and documented.

Baseline (day 0): randomization

Treatment period (day 0~70 ± 1 day): patient attends the study location on days 28, 56, and 70.

Post-treatment follow-up (day 98 ± 3 days): patient attends the study location for a follow-up visit to assess outcomes including safety and survival.

### Patient selection and inclusion criteria

A total of 84 subjects will be enrolled. Inclusion criteria are as follows:

- 18 years ≤ age ≤ 70 years
- Patients eligible for ILD, including CTD-ILD, interstitial pneumonia with autoimmune features (IPAF), and IPF
- ≥ 40 mm on the Cough Severity Visual Analogue Scale (VAS) at the screening period
- No diagnosis or history of upper airway cough syndrome, cough variant asthma, or eosinophilic bronchitis; no cough relief by treating these causes
- No contraindications to gabapentin treatment
- Able to read, understand, and give written informed consent

### Exclusion criteria

Subjects meeting any of the following criteria will not be eligible to participate:

- Pregnant or lactating women or those unwilling to sign the informed consent form
- Smoking within the past 2 years
- Those who have used gabapentin within 2 months
- History of respiratory tract infection within 8 weeks
- Patients with acute exacerbation of interstitial pneumonitis (AEIP)
- Cough caused by GER
- Respiratory failure suggested by arterial blood gas analysis
- Severe heart, liver, kidney, other vital organs, blood, and endocrine system diseases
- Current active infection, glucocorticoid therapy, or immunosuppressive therapy
- Positive hepatitis B virus surface antigen or positive hepatitis C antibody
- Mental illness or other reasons for inability to cooperate with treatment
- Allergies including multiple drug allergies
- Other conditions deemed by the investigator to be unsuitable for participation in the research

### Elimination criteria

Case elimination is a prudent and scientific approach to trial data and information gathered by blinded researchers, after discussion between the study leader, data manager, statistical analysis leader, and the sponsor.

The following criteria for elimination are key to determining the data set for statistical analysis of trial endpoints:

- Patients who were included in error despite not meeting inclusion criteria, or were randomly assigned but do not meet the randomization criteria
- Patients who meet the inclusion and randomization criteria but do not meet the exclusion criteria, do not take the trial drugs after inclusion, or fail to attend for follow-up, affecting the evaluation of the effectiveness and safety
- Patients taking a combination of medications in violation of the protocol, especially those with impact on factors related to effectiveness and safety
- Patients who become unmasked (unblinded) during the trial.

### Sample size estimate

A prospective statistical power calculation based on the results of pre-experiment and published data (gabapentin for refractory chronic cough: a randomized, double-blind, placebo-controlled trial) [17] indicated that LCQ scores were 15.8 ± 3.1 and 13.2 ± 3.9 in patients treated with gabapentin for cough and control patients, respectively. Therefore, a minimum of 33 patients per group would be required to provide 80% power for a 5% two-sided test. Including the predicted 20% attrition rate, the final allocation numbers were 42 patients per group. Given the number of individuals treated with gabapentin at our unit, this number is feasible within the planned trial timelines.

### Randomization, sequence generation, and allocation concealment

In order to participate, patients meeting all of the above criteria must be randomized, discontinue other medications for respiratory diseases during the introductory period, and complete the screening and introduction period with good compliance. A randomized coding table with consecutive numbers (01 to 84) will be generated in SPSS statistical software for randomized allocation to groups. Patients meeting the criteria for randomization will be assigned random codes in strict order and receive the drug assigned by the code. This assignment will be conducted exclusively by one investigator who is not involved in the subsequent related interventions and follow-up of patients.

### Blinding

Participants and researchers in the trial will be blinded to the generation of randomized numbers, numbering of experimental drugs, enrollment of subjects for administration, recording and evaluating the trial results, and monitoring the trial process and data management. In order to enable smooth implementation of the double-blind trial, a double-blind single simulation technique is used to ensure that the placebo provided is identical to the simulated gabapentin capsules in terms of dosage form, appearance, properties, and odor, and does not contain the active ingredient. Both gabapentin capsules and placebo are labeled with the name “gabapentin,” indicating the dosage, method of storage, and drug number. The label will be unblinded only if the subject has a serious adverse event (SAE) or requires emergency resuscitation and investigators need to know which treatment he or she is receiving.

### Interventions

The gabapentin group is treated with gabapentin, while the control drug treatment group is given a placebo in the same package and at the same dose. Gabapentin and placebo are administered as follows: the starting dose will be 100 mg per dose, 3 times per day; the dose is increased by 100 mg every 3 days thereafter until 300 mg per dose, 3 times per day, or the onset of intolerable drug side effects. After reaching the maximum dose, the treatment is maintained for 70 days (10 weeks). Gabapentin and placebo are then discontinued, and patients are again assessed for symptoms after 4 weeks of discontinuation.

### Reduction or discontinuation

The reduction or discontinuation of the experimental drug during the trial will be avoided. However, the investigator may consider dose reduction, suspension, or discontinuation if one of the following occurs:

- The occurrence of SAEs related to the trial drug
- Occurrence of adverse reactions (AR) with the test drug that are not effectively relieved by symptomatic treatment
- Complication/new onset of other diseases which the test drug may aggravate
- Significant abnormalities in safety-related laboratory indicators
- Other circumstances that the investigator considers necessitate reduction, suspension, or discontinuation of the dose

### Concomitant medication

Both groups are given prednisone combined with the classical drug cyclophosphamide (CTX) sequential azathioprine (AZA) treatment.

### Prohibited concomitant medication

Patients will be required to avoid combining drugs where possible, especially combining medications with a significant impact on effectiveness and safety. However, in the case of adverse events (AEs), deterioration of the original condition, or complication/new onset of other serious diseases during the trial, the investigator will promptly combine the drugs and actively carry out rescue treatment. If the subject meets the withdrawal criteria, the investigator will make proper arrangements for withdrawal from the trial. All concomitant medication (including prescription and over-the-counter medications) will be recorded in the case report form (CRF) during the trial, including details of the drug name, single dose, frequency of administration, routine of administration, reason for administration, start and end time of administration, and any changes to dosage. In particular, combined drug use in the event of AE will be recorded and reported in a timely fashion.

### Discontinuation or withdrawal of study subjects

Subjects may voluntarily withdraw from the trial at any time for any reason, and the investigator may also discontinue the participation of any subject in the trial for a variety of reasons, primarily including safety concerns or protocol violations.

Subjects should withdraw if any of the following occur:

Subjects are unwilling to continue:

- Subjects discontinue the drug on their own initiative and do not wish to continue the trial due to poor efficacy or ineffectiveness, or other reasons
- Missed follow-up visits due to changes in work and living environment or accidents (if traffic accidents, deaths, or fractures, follow-up visits should be made in a timely fashion and any causal relationship with the test drug will be determined)
- The subject withdraws informed consent
- The subject is lost to follow-up

SAE, or more serious allergic reactions:

- The subjects are deemed by the investigator unsuitable to continue to use the experimental drugs and/or must be monitored on the basis of safety and ethical considerations due to significant abnormalities in safety-related laboratory indicators, or the occurrence of SAEs, or more severe allergic reactions associated with the study drug
- Emergency unblinding due to SAE requiring emergency resuscitation

Pregnancy; deterioration of the original condition or complication/new onset of other serious diseases:

- The original disease worsens or the patient could not tolerate it during the trial, and the investigator judges that it is inappropriate for the patient to continue participating in the trial
- Due to complications/new onset of other serious illnesses during the trial, such as tuberculosis or tumors, the investigator judges that it is inadvisable for the patient to continue the trial

Serious violations of the test protocol:

- Serious violations include patients not meeting the selection criteria and/or meeting the exclusion criteria, or patients not signing the informed consent
- Other serious violations include the patient’s inability to undergo the required examinations as specified in the protocol, or unplanned pregnancy during the trial

### Exit procedures

- If patients have already signed the ICF, but not been randomized, or if AE or SAE occurs during the introductory period, the investigators will document the patient’s demographic characteristics and reasons for withdrawal.
- If the subjects withdraw midway through the trial, the researcher will try to complete the examination and assessment of outcome indicators within 2 weeks after withdrawal (preferably before the start of other treatment). Investigators will contact patients who are lost to follow-up. The date of the last dose and the reason for early discontinuation of the trial for patients who terminate the experiment early will be recorded in the CRF.

Subjects who withdraw from the trial after randomization cannot be replaced; the randomization number is uniquely associated with each subject and cannot be reused.

### Efficacy measurement procedures

Observations include changes in cough symptom score, LCQ, Gerd Q, HARQ, and capsaicin cough thresholds C2 and C5 at baseline, 4 weeks, 8 weeks, 10 weeks, and 14 weeks of treatment. Any drug side effects will be recorded. Assessment of ILD activity scores (chest CT, lung function, symptoms) will be made at baseline and after 14 weeks of treatment (i.e., 4 weeks off the drug).

Assessments used for case diagnosis and follow-up observation include pulmonary function, bronchial excitation test, multichannel esophageal impedance-pH monitoring, induced sputum, and capsaicin cough sensitivity test. All techniques will be performed in our respiratory department according to the methods established by relevant international and national guidelines. All biological samples used for research will be collected, processed, and analyzed according to the specific research procedures of the research unit to achieve accurate correlation with clinical data.

### Statistical analysis

Statistical analysis will be conducted using SPSS 22.0 statistical analysis software. All statistical tests will be performed using two-tailed tests, and *P* ≤ 0.05 will be considered statistically significant. Measurement data will be described using mean and standard deviation, and paired *t*-tests will be used to compare pre- and post-treatment differences. Count data will be statistically described by frequency (composition ratio), and changes before and after treatment will be tested by *χ*^2^ test or other non-parametric tests.

### Patient and public involvement

Patients will participate in the study voluntarily. They are not involved in the design and dissemination plans of the study. Some of the outcome indicators will be patient-reported, including cough symptoms, LCQ, Gerd Q, HARQ, and others, and timely feedback on safety (adverse effects) during the treatment.

## Outcomes

### Primary outcome measure

The primary outcome is change in refractory cough associated with ILD, indicated by cough symptom scores. Efficacy criteria are as follows: complete disappearance of cough indicates resolution; 50% or more reduction in the sum of daytime and nocturnal cough symptom scores indicates effective treatment; < 50% reduction in the sum of daytime and nocturnal cough symptom scores or worsening indicates ineffective treatment.

### Secondary outcome measures

- Change in quality of life due to cough. Ending indicator: change in the scores of LCQ.
- Change in gastroesophageal reflux. Ending indicator: change in the scores of Gerd Q and HARQ.
- Cough sensitivity. Ending indicator: changes in capsaicin cough sensitivity (C2 values and C5 values). When the same patient is followed up, the same time period is chosen to record the capsaicin cough sensitivity test. Capsaicin cough sensitivity test is performed within 1 h of the cough count.
- Safety and tolerability of gabapentin. Ending indicator: AE/SAE, vital signs, liver and kidney function, and fecal occult blood test.

### Assessment of adherence

Adherence will be assessed as actual medication dose/theoretical medication dose × 100%, where actual dose = total amount of medication dispensed − (total amount of remaining return + total amount of lost).

Theoretical dose = individual dose × number of doses

Good adherence: 80 to 120%; poor adherence: 120%

### Data management and data checking

This study will use paper storage and Excel input for preservation and analysis. Data entry personnel will have rights to access for data entry, modification, and challenge resolution. Researchers will have permission for specific modification, browsing, challenge resolution, and review permissions. Monitors have permissions to browse, send/close challenges, freeze data, and lock. The researcher will maintain data ensuring that it is accurate, complete, and timely. The original documents and medical records will be clear, detailed, and easily identifiable by those participating in this trial.

### Protocol amendments

Reports on data monitoring and security will be submitted to the ethics committee on a 3-monthly basis. The researcher will submit any necessary amendment application to the ethics committee for any change in the principal investigator, the clinical study protocol, informed consent, recruitment materials, or other modification.

### Assessment of safety

Examination of the occurrence of AEs and clinical endpoints will begin with randomized groups and will continue focusing on individual patients until they complete follow-up at 14 weeks. At each visit, the researcher will conduct a safety assessment and will specifically review the clinical history related to the occurrence of adverse or SAEs and any causal relationship with the study drug to evaluate the AEs severity. Details of AEs and clinical events related or not to the trial drug will be captured in the eCRF. All AEs will be tracked until resolved.

### Serious adverse event (SAE) reporting and adverse event (AE) reporting

AE reporting begins on signing the ICF. All AEs related or not to the trial drug must be recorded in the corresponding parts of the CRF. All AEs should be described by concise medical terminology, including their:

- Name
- Start and end time
- Severity
- Measures taken due to AE
- Regression
- Any correlation between AE and test drug

During this study, if any SAE occurs, the researcher will report it to CFDA, the health administration department, the relevant provincial or autonomous region or municipality drug regulatory authority, the ethics committee of the investigator’s research center, and the sponsor within 24 h. The sponsor is responsible for SAE safety inspections, will ensure that the research center completes all SAE reports in compliance with the requirements of regulatory agencies and local regulations, and will report the SAE to the corresponding regulatory agency in accordance with these requirements.

### Ancillary and post-trial care

The sponsor will pay reasonable travel expenses for participation in this study. If subjects suffer an injury during study participation or an adverse event during drug treatment, they will contact their study physician and will receive prompt treatment. In the case of injury that is causally related to the study or the drug used in this trial, the sponsor will bear the medical costs and provide the patient with appropriate financial compensation in accordance with the relevant national laws and regulations. Patients retain all their legal rights and interests during participation.

### Ethics and dissemination

Before starting the trial, the protocol and relevant documents are submitted to the appropriate ethics committee for approval. The consent/approval signed by the committee will be kept in the investigator’s document file. After the formal initiation of the trial, before the screening of subjects, the investigator will give informed consent instructions to each target patient attending the clinic. The approved patient information consent form is available as Additional file 1. The ICF will be signed voluntarily by the subject, their legal representative, or guardian on the premise that the patient fully understands the trial process and agrees to participate in the trial. The study process and the acquisition of informed consent will comply with the ethical principles of the Declaration of Helsinki, the relevant GCP requirements, and the laws related to drug and data protection of China. Finally, the results of the study will be published in the form of a paper.

## Discussion

Cough is the most common and most difficult symptom to treat in ILD. In addition, the clinical efficacy of refractory cough treatment remains unsatisfactory due to the complex and unknown disease etiology and numerous adverse drug reactions. This trial aims to provide a new therapeutic tool by evaluating the efficacy of gabapentin in the treatment of refractory cough associated with ILD by assessing the improvement of poor quality of life due to cough and cough sensitivity, as well as the disease activity score. It will also confirm that improving airway sensitivity is a new opportunity to treat cough symptoms in patients with ILD and provide additional insight into the etiology of refractory cough.

### Trial status

The project version number of this research: (V1.0) 2020.12.02. The study is currently actively recruiting in the CN.

## Supplementary Information

**Additional file 1.** Informed Consent Form.

## Data Availability

Not applicable, as data is not yet available. When the experiment is completed, the complete protocol and metadata will be available upon reasonable request.

## Abbreviations

ILD: Interstitial lung disease
CTD: Connective tissue disease
IPF: Idiopathic pulmonary fibrosis
CHS: Cough hypersensitivity syndrome
GER: Gastroesophageal reflux
GERD: Gastroesophageal reflux disease
IPAF: Interstitial pneumonia with autoimmune features
AEIP: Acute exacerbation of interstitial pneumonitis
LCQ: Leicester Quality of Life Questionnaire
Gerd Q: Gastroesophageal Reflux Disease Questionnaire
HARQ: Hull Airway Reflux Questionnaire
Chest CT: Chest computer tomography
CTX: Cyclophosphamide
AZA: Azathioprine
