# Supplementary material for: The efficacy of gabapentin for the treatment of refractory cough associated with interstitial lung disease: study protocol for a randomized, double-blind and placebo-controlled clinical trial
Source: Trials. 2022 Feb 21;23:165. doi: 10.1186/s13063-022-06059-5 (PMC8862465; doi:10.1186/s13063-022-06059-5)
Supplement: Supplementary file 1 — Additional file 1. Informed Consent Form. [file 13063_2022_6059_MOESM1_ESM.docx]

**Informed Consent Form**

**Project name:** A randomized, double-blind, placebo-controlled clinical study of gabapentin for the treatment of refractory cough associated with interstitial lung disease

**Version: 1.0**

**Research unit:** Shanghai Tongji Hospital

**Principal investigator:** Li Yu, Xianghuai Xu, Xuan Wang

You are being invited to participate in a clinical research study. This informed consent form gives you some information to help you decide whether to participate in this clinical study. Please read it carefully and ask the investigator in charge of the study if you have any questions.

Your participation in this study is voluntary. This study has been reviewed by our Institutional Ethics Review Board.

If you have any questions or concerns about this study, you may contact the study physicians: Xuanghuai Xu and Xuan Wang at 021-66111065 or 021-66111086.

If you have questions about the rights of the subjects, you can contact the Ethics Committee of Shanghai Tongji Hospital at 021-66111243 or email: tongjilunli2012@163.com.

A total of 84 subjects were planned to be included in this study, and one hospital participated in this study.

**1.Aim and objective**

The primary aim of this study is to investigate whether gabapentin could improve cough symptoms in patients with ILD through a randomized, double-blind, placebo-controlled clinical study. The secondary is to explore the potential mechanism of gabapentin to improve cough symptoms in ILD.

**2.Background**

Interstitial lung disease (ILD) is a collective term for a group of acute and chronic lung diseases affecting the airways, lung parenchyma and pulmonary vascular system, with varying degrees of airway inflammation and fibrosis. There are two main categories of ILD: the first caused by connective tissue disease, environmental exposures, and medications, and there are seven main types of the latter, the most common of which is idiopathic pulmonary fibrosis (IPF). The clinical manifestations of ILD are similar and include dyspnea, cough, and pulmonary hypertension, among which cough is the most common and difficult to treat symptom of ILD.

Studies on cough associated with ILD are relatively few and uncritical, and some drugs may be effective, but there are problems with unknown specific mechanisms and high incidence of adverse drug events, and further studies are still needed. Pirfenidone and nidanib have been used as antifibrotic therapy for ILD, but there is still a lack of support from larger clinical trials. Corticosteroids have been shown to reduce cough, but the "triple therapy" of corticosteroids combined with azathioprine and n-acetylcysteine for IPF has resulted in increased mortality compared with the placebo group, so the use of corticosteroids should be limited to patients with possible exacerbations of ILD or patients with coexisting asthma or eosinophilic bronchitis. A single-center study of thalidomide for IPF-associated cough showed that thalidomide significantly improved patients' quality of life; however, the significant side effects of thalidomide have led to its not being recommended for IPF-associated cough.

The complexity and variety of mechanisms pose a great challenge in the search for the etiology and subsequent treatment of cough in patients with ILD. The mechanisms underlying cough in patients with ILD are complex and may be related to airway inflammation, sensitization or pulmonary fibrosis, interstitial lung disease drug therapy, infection, and concomitant diseases (e.g., gastroesophageal reflux, upper respiratory tract disease, and asthma). Multiple clinical studies have shown that cough symptoms persist in patients with ILD alongside anti-inflammatory and anti-fibrotic therapy, suggesting that improving airway sensitivity may be a new opportunity to treat cough symptoms in patients with ILD.

The concept of CHS has the potential to point to new research directions for the development of more effective therapeutic agents and the improvement of the diagnosis and treatment of refractory cough in the future. The concept of CHS has been recognized by peers.

Gabapentin inhibits neurotransmitter release by specifically binding to the α2δ subunit of voltage-gated calcium channels in the brain, primarily for the treatment of epilepsy and neuropathic pain. Because patients with chronic cough have a central hypersensitivity similar to that of neuropathic pain, gabapentin is now also used to treat refractory chronic cough. Well-designed clinical trials have demonstrated that gabapentin at therapeutic doses gradually increasing from 300 mg/d to 1800 mg/d for 8 weeks significantly improves symptoms and quality of life in patients with refractory cough. Gabapentin is also recommended for the treatment of refractory cough in both the new Chinese and US cough guidelines. Our previous study and other studies have shown that gabapentin is effective in treating patients with refractory cough at around 57%.

In addition, gastroesophageal reflux is one of the main causes of cough in patients with ILD. For GERD-associated cough, Madanick et al. combined 300 mg-900 mg/d of gabapentin with a control drug to treat GERD-associated cough, which reduced cough symptoms by at least 50% in 75% of patients. Our previous clinical study also confirmed that gabapentin as an add-on therapy to standard anti-reflux drug therapy was effective in controlling cough symptoms in 57% of refractory GERD-associated cough, with similar efficacy to another neuro factor modulator, baclofen, but with fewer side effects, better patient tolerance, and mostly within 1 week of onset action. It was confirmed that the anti-reflux measures with gabapentin at the beginning of the treatment period are a fast-acting treatment option. In conclusion, the use of gabapentin for the treatment of refractory cough caused by ILD has a good clinical application.

**3. Inclusion and exclusion criteria (who can participate in this study?):**

You may participate in this study if you meet the following requirements.

- 18 years ≤ age ≤ 70 years;
- Patients eligible for ILD, including CTD-ILD, interstitial pneumonia with autoimmune features (IPAF), IPF;
- ≥40mm on the Cough Severity Visual Analogue Scale (VAS) at the screening visit;
- The upper airway cough syndrome, cough variant asthma and eosinophilic bronchitis cannot be diagnosed on the basis of medical history and laboratory examinations, or the cough cannot be relieved and only partially relieved by treating these causes;
- No contraindications to gabapentin treatment;
- Able to read, understand and give written informed consent.

You may not participate in this study if you meet the following requirements.

- Pregnant or lactating women or those who refuse to sign the informed consent form;
- Smoking or quit smoking ≤ 2 years;
- Those who have used gabapentin within 2 months;
- History of respiratory tract infection within 8 weeks;
- Patients with the acute exacerbation of interstitial pneumonitis (AEIP);
- The cough caused by GER;
- Arterial blood gas analysis suggests respiratory failure;
- Severe heart, liver, kidney, other vital organs, blood and endocrine system diseases;
- Aggravated by the presence of active infection, glucocorticoids therapy and immunosuppressive therapy;
- Positive hepatitis B virus surface antigen, or positive hepatitis C antibody;
- Mental illness or other reasons for inability to cooperate with treatment;
- People with allergic body or multiple drug allergies;
- Other conditions deemed by the investigator to be unsuitable for entering the research.

**4. Research process (what do you need to do if you participate in this study?):**

If you agree to participate in this study, we will number each participant, create a study file, and divide them into gabapentin treatment and placebo groups by a randomized, double-blind method. The gabapentin group is treated with additional gabapentin, while the control drug treatment group is given the same package and dose of placebo. Gabapentin and placebo are administered as follows: the starting dose was 100 mg per dose, 3 times a day; the per dose is increased by 100 mg every 3 days thereafter until 300 mg per dose, 3 times a day, or the onset of intolerable drug side effects. After reaching the maximum dose, the treatment is maintained for 70 days (10 weeks). Gabapentin and placebo are then discontinued, patients are again assessed for symptoms after 4 weeks of discontinuation. We will examine you at weeks 0, 4, 8, 10 and 14, evaluate your symptoms and hope to have your understanding and assistance.

**5. Risks and discomforts:**

Allergy, dizziness, drowsiness, peripheral edema, etc. may occur during the course of a trial with gabapentin or placebo.

If the subject experiences any discomfort, new changes in condition, or anything unexpected during the study, whether or not related to the drug/test, the subject's primary care physician should be contacted promptly, and the physician will make a judgment call and provide medical treatment.

**6. Possible benefits (what will this study do for me):**

If you are in the trial drug group, the trial drug may cure the disease or slow the progression of the disease, but we (the investigators) cannot guarantee this. If you are in the placebo control group, there may be no direct benefit, but it can contribute to medical progress.

**7. Possible additional costs or burdens (will I have to pay anything to participate in this study?):**

There are no additional expenses for you to participate in this research project. The cost of gabapentin used in this study is covered by the research grant.

**8. Compensation received for participating in the study:**

We will pay your reasonable travel expenses for participation in this study, CNY 50 for each clinical visit, for a total of 3 visits.

**9. Medical treatment and compensation for injuries (what to do if you are injured while participating in the study):**

If you suffer an injury during study participation or an adverse event during drug treatment, please contact your study physician and you will receive prompt treatment; any injury that is causally related to the study or the drug used in this trial, the sponsor will bear the medical costs and give you appropriate financial compensation in accordance with the relevant national laws and regulations.

Even if you have signed this informed consent form, you retain all your legal rights and interests.

**10. As a research subject, you have the following responsibilities:**

Provide truthful information about your medical history and current medical condition; tell the study doctor about any discomfort you experience during this study; refrain from taking restricted medications, foods, etc.; and tell the study doctor if you have participated in other studies recently or are currently participating in other studies.

**11. Privacy and confidentiality:**

If you decide to participate in this study, we will make every effort to protect your personal privacy to the extent permitted by law. Any public reporting of the results of this study will not disclose any personal information about you. The physician in charge of the study and other researchers will use your medical information to conduct the study. This information may include your name, address, telephone number, medical history, and information obtained at the time of your study visit. Information that identifies you will not be released to members outside the study team unless your permission is obtained. All study members and study sponsors are required to keep your identity confidential. Your file will be kept in a locked file cabinet and will be accessible only to the researcher. To ensure that the research is conducted in accordance with regulations, members of the government administration or ethics review committee will have access to your personal information at the research unit, as required. When the results of this study are published, no personal information about you will be disclosed.

We will contact you promptly about any meaningful new developments or new medical information related to your health during the study, such as suggesting that you undergo tests to determine such new information. I will also keep you informed of any new information that may affect your choice to continue in the study.

**12. Are there any other treatments available and what are the advantages and disadvantages of the treatments used in this study?**

Standard treatment: None at this time.

Approach 1: Pirfenidone and Nidanib - primarily targeted for antifibrotic therapy, may be effective for cough, but specific mechanisms and clinical support are lacking.

Approach 2: Biologic agents - expensive and with uncertain efficacy.

**13. Subject rights:**

Participation in the study is entirely voluntary. You may refuse to participate in the study, or withdraw from the study at any time during the study, and your data will not be included in the study results. None of this will affect your relationship with your physician. Any medical treatment or rights you may have will not be affected by this.

The study physician may terminate your participation in the study if you require other treatment, if you fail to comply with the study plan, if you have a study-related injury, or for any other reason.

**Informed Consent Form Signature Page**

I have read this informed consent form and have discussed and asked questions about this study with my doctor. I have been given a detailed explanation of the purpose of the study, the study process, the possible risks and benefits, and all my questions have been answered, and I understand that participation in this study is voluntary.

I acknowledge that I have had sufficient time to consider this, including the possible risks of participating in the study. I am aware that I can consult with my physician at any time for further information, that I can withdraw from the study at any time without discrimination or reprisal, that my medical treatment and benefits will not be affected by withdrawal from the study, and that it would be in my interest and that of the study as a whole to inform my physician of any changes in my condition and to complete appropriate physical and physical examinations if I withdraw from the study, particularly for treatment reasons. If I need to take any other treatment as a result of a change in my condition, I will seek prior advice from my physician or tell him/her truthfully afterwards.

I am voluntarily participating in this study. I give my consent to the investigator, sponsor, health administration supervisory authority/drug and food regulatory authority, and ethics committee to access my study data.

I will be given a signed and dated copy of the informed consent.

Subject's name: _____________. Proxy's name: _____________.

Subject's signature: __________. Proxy's signature: _____________.

Date: _____________. Date: _____________.

(Note: Signature of witness required if subject is illiterate and signature of agent required if subject is incapacitated)

I have accurately informed the subject of this document and he/she has accurately read this informed consent form and certify that the subject has had the opportunity to ask questions. I certify that he/she has given voluntary consent.

Name of Investigator: ________________________

Investigator's signature: _________________________

Date: _________________________
